# Intestinal motility distal of a deviating ileostomy after rectal resection with the construction of a primary anastomosis: results of the prospective COLO-MOVE study

**DOI:** 10.1007/s00384-020-03651-6

**Published:** 2020-06-05

**Authors:** T. A. Burghgraef, F. J. Amelung, P. M. Verheijen, I. A. M. J. Broeders, E. C. J. Consten

**Affiliations:** 1 Department of Surgery, Meander Medical Center, Maatweg 3, 3813 TZ Amersfoort, the Netherlands; 2 Department of Surgery, University Medical Center Groningen, Hanzeplein 1, 9713 GZ Groningen, the Netherlands; 3 Department of Surgery, University Medical Center, Utrecht, the Netherlands

**Keywords:** Rectal neoplasms, Gastrointestinal motility, Bowel preparation

## Abstract

**Purpose:**

No consensus exists regarding the use of preoperative bowel preparation for patients undergoing a low anterior resection (LAR). Several comparative studies show similar outcomes when a single time enema (STE) is compared with mechanical bowel preparation (MBP). It is hypothesized that STE is comparable with MBP due to a decrease in intestinal motility distal of a newly constructed diverting ileostomy (DI).

**Methods:**

In this prospective single-centre cohort study, patients undergoing a LAR with primary anastomosis and DI construction were given a STE 2 h pre-operatively. Radio-opaque markers were inserted in the efferent loop of the DI during surgery, and plain abdominal X-rays were made during the first, third, fifth and seventh postoperative day to visualize intestinal motility.

**Results:**

Thirty-nine patients were included. Radio-opaque markers were situated in the ileum or right colon in 100%, 100% and 97.1% of the patients during respectively the first, third and fifth postoperative day. One patient had its most distal marker situated in the left colon during day five. In none of the patients, the markers were seen distal of the anastomosis.

**Conclusion:**

Intestinal motility distally of the DI is decreased in patients who undergo a LAR resection with the construction of an anastomosis and DI, while preoperatively receiving a STE.

## Introduction

In colorectal resections, bowel preparation is used to clean the colon of faeces, thereby preventing the passage of faeces across the anastomosis, aiming to reduce anastomotic leakage. Mechanical bowel preparation (MBP) can safely be omitted in colon resections, despite a recent suggestion of using the combination of oral antibiotics and MBP [1, 2]. However, discussion remains regarding the use of bowel preparation in rectal cancer surgery due to the high risk of anastomotic leakage [2].

MBP is not harmless: hypovolemia, electrolyte imbalances, renal failure and discomfort for the patient have been reported [3, 4]. In addition, MBP might not reduce gut microbial flora, but liquefy faeces, thereby increasing the risk of spillage and intra-abdominal contamination [5]. Despite these adverse effects, omission of colon preparation does not seem feasible, since a randomized controlled trial showed MBP to be favourable over no bowel preparation in rectal cancer patients [3].

Single time enema (STE) has been suggested as an alternative for bowel preparation, as it is less burdensome and not associated with the above-mentioned complications associated with MBP [2]. Two studies show similar (infectious) outcomes when STE is compared with MBP in patients who underwent a low anterior resection (LAR) with the construction of a primary anastomosis and diverting ileostomy (DI) [6, 7].

This indicates that, even though faeces remain distal of the DI following preparation with an STE, it does not cause an increase in complication rate. Leading to the hypothesis that, intestinal motility distal of a newly constructed DI is decreased or even halted in the early postoperative phase. This prevents faeces to pass the anastomosis, resulting in comparable (infectious) complications, while only administering a STE.

Intestinal motility distal of a DI has already been shown to be decreased in patients that underwent a rectal resection with pre-operative MBP. However, it remained unclear whether the decrease in intestinal motility was due to MBP and the subsequent lack of faeces or a direct consequence of the DI [8]. This study aims to verify that intestinal motility is decreased after rectal resection and construction of a DI, when a STE is given as pre-operative bowel preparation.

## Materials and methods

A prospective single-centre study was performed assessing intestinal motility distal of a DI in patients who underwent a LAR with the subsequent construction of a primary anastomosis and DI.

### Patients

Study candidates were consecutively recruited and included if they (1) were older than 50 years, (2) had rectal carcinoma, (3) underwent an elective rectal resection with primary anastomosis, (4) received a DI, (5) received pre-operative STE and (6) gave informed consent. Exclusion criteria were (1) previous colonic resection, (2) known gastro-intestinal motility disorder, (3) allergy for gelatine or plastic or (4) a contra-indication for the use of rectal enema. Patients younger than 50 years were excluded to minimize the possible long-term consequences of additional radiation during the study. Patients with gastro-intestinal motility disorder were excluded since this would affect postoperative motility of the intestine.

### Procedures

All study participants received a ‘low’ STE (Microlax, Johnson & Johnson, Amersfoort, the Netherlands) 2 h pre-operatively. A robot-assisted low anterior resection was performed. Following completion of a primary anastomosis, a DI was constructed. Twenty-four radiopaque markers were placed in the efferent loop of the DI.

Transit of the markers trough the colon was determined by plain abdominal X-rays taken at the first, third, fifth and seventh day postoperatively. Additional abdominal imaging, performed during follow-up, was included in the analysis. The location and number of markers were registered (terminal ileum/right colon, left colon, (neo)rectum or excreted). Intestinal motility was defined as a change in location of the most distal marker. Furthermore, patient characteristics, 30-day morbidity and mortality and the occurrence of faeces excreted through the rectum as observed by nursing staff or the patient were determined.

### Statistical analysis

Statistical analysis was done using R version 3.5.1. Continuous variables were expressed as mean and standard deviation (SD), or median and interquartile range (IQR), depending on the distribution. Categorical data were presented as absolute numbers and percentages.

## Results

### Patient characteristics

Between February 2016 and March 2019, 99 eligible patients were approached and 87 patients gave informed consent. However, 37 patients did not receive a DI or did not undergo a LAR. Furthermore, markers were inadequately placed in nine patients and two patients refused to undergo the postoperative X-rays, resulting in 39 included patients.

Patients had a median age of 65, a median BMI of 24.4, 66.7% was male, 89.7% of the patients were scored as ASA I-II and 10.3% as ASA III. The tumour was located between 0 and 5 cm of the anal verge in 23.1%, and between 5 and 10 cm in 69.2% of the patients. Neoadjuvant (chemo)radiation was received by 52.0% of the patients.

### Abdominal imaging

In patients who underwent abdominal imaging during day one and day three, 100% of the markers were seen. In patients who underwent imaging during day five, one patient showed that the most distal marker migrated to the left colon (Table 1).

**Table 1:** Location of most distal markers

Time	Imaging	Marker location	
Day 1 (*n*, %)	37/39 (94.9 %)	Right colon	37 (100 %)
		Left colon	0 (0 %)
Day 3 (*n*, %)	36/39 (92.3%)	Right colon	36 (100 %)
		Left colon	0 (0 %)
Day 5 (*n*, %)	34/39 (87.2 %)	Right colon	33 (97.1 %)
		Left colon	1 (2.9 %)
Day 7 (*n*, %)	15/39 (38.5 %)	Right colon	14 (93.3 %)
		Left colon	1 (7.1 %)
3 months (*n*, %)	21/39 (53.8 %)	Right colon	8 (38.1 %)
		Left colon	4 (19.0 %)
		(Neo) rectum	1 (4.8 %)
		Excreted	8 (38.1 %)

Rectal excretion of faeces postoperatively, with the DI in situ, was reported in 28.2% of the patients. In two patients, this occurred within 14 days postoperatively: At the fourth and the eleventh postoperative day. The patient reporting rectal excretion of faeces on the fourth postoperative day had an anastomotic leakage with faecal peritonitis, but without marker movement on the abdominal X-rays.

### Postoperative outcome

No 30-day mortality was observed. Thirty-day major morbidity, defined as Clavien-Dindo classes III and IV, was seen in six patients (15.4%). Five had an anastomotic leakage. This was treated conservatively with antibiotics in two patients, two patients required radiological drainage of a presacral abscess and one patient had to undergo a re-laparotomy with the construction of a new colostomy. A completely dehiscent anastomosis was seen during the re-laparotomy, with stapler remnants and faecal peritonitis. Marker movement in patients with an anastomotic leakage (*n* = 5) was absent.

## Discussion and conclusion

This study aimed to provide information regarding the intestinal motility distal of a newly constructed ileostomy in patients undergoing a LAR with primary anastomosis, while pre-operatively receiving a STE. The results suggest that intestinal motility distal of the DI is absent or decreased in the majority of patients at least up until the fifth postoperative day.

Ali et al. found intestinal motility distal of a DI to be decreased in patients who underwent a LAR with the construction of a DI, while receiving MBP pre-operatively [8]. However, since these patients underwent MBP, it was unclear whether the halted intestinal motility was caused by the lack of faeces present in the colon or due to the construction of a deviating ileostomy. Huang et al. reported decreased motility in ten patients following LAR with primary anastomosis and DI construction while pre-operatively receiving a STE [9]; however, the sample size was low. Our study confirms the suggestion of Huang et al. and Ali et al. In addition, we show that patients preoperatively subjected to STE show decreased motility as well.

The mechanisms responsible for the decrease in intestinal motility remain unclear. Intestinal motility consists of segmental propagated contractions, which are arbitrarily divided into low-amplitude propagated contractions (LAPC) and high-amplitude propagated contractions (HAPC) [10]. The latter play a significant role in the oro-aboral shift of colonic content and require an intact enteric nervous system. In addition, it has been suggested that intraluminal continuity is necessary for intestinal motility [11, 12]. Perhaps HAPCs are interrupted due to lack of intraluminal continuity.

Although intestinal motility distal of a DI is decreased, certain findings should be taken into account. First, this study is not a comparative study; therefore, our results are only applicable for patients who underwent rectal resection with primary anastomosis and DI, while preoperatively receiving a STE.

Secondly, the patient who had an anastomotic leakage with faecal peritonitis did not show intestinal motility on the plain abdominal X-rays, although faeces was excreted anally four days postoperative. The presence of faecal content in the abdominal cavity is in contrast with our hypothesis that intestinal motility distal of a DI is halted following DI construction. Since none of the markers migrated in this patient, perhaps the total dehiscent anastomosis facilitated intra-abdominal spillage of faecal content, especially in combination with gravitational force. More importantly, we used ‘low’ STE’s which can only clean the rectum and the distal sigmoid. ‘High’ STEs might be necessary to prevent (passive) passage of faeces across the anastomosis by gravitational force.

Despite these limitations, the majority of patients did not show signs of intestinal motility distal of the DI on abdominal X-ray. Subsequently, this study suggests a decrease or even a halt in intestinal motility distal of a DI in patients receiving a DI in the context of a LAR with primary anastomosis. Since bowel preparation is aimed at prevention of faecal passage across the anastomosis, these results suggest that a ‘high’ STE might be sufficient with decreased intestinal motility distal of a DI. However, a comparison between STE and MBP in patients receiving a LAR with primary anastomosis and a DI is necessary to confirm this.

## Data Availability

Anonymized patient data is available.
